# Hot Deformation Behavior of Homogenized Mg-13.5Gd-3.2Y-2.3Zn-0.5Zr Alloy via Hot Compression Tests

**DOI:** 10.3390/ma11112282

**Published:** 2018-11-14

**Authors:** Zhimin Zhang, Zhaoming Yan, Yue Du, Guanshi Zhang, Jiaxuan Zhu, Luying Ren, Yiding Wang

**Affiliations:** 1 School of Material Science and Engineering, North University of China, Taiyuan 030051, China; nuc_duyue@126.com (Y.D.); s1403035@st.nuc.edu.cn (G.Z.); s1603041@st.nuc.edu.cn (L.R.); 2 College of Mechatronics Engineering, North University of China, Taiyuan 030051, China; s1501046@st.nuc.edu.cn; 3 State Key Laboratory of Solidification Processing, Northwestern Polytechnical University, Xi’an 710072, China; npu_ydwang@163.com

**Keywords:** Mg-Gd-Y-Zn-Zr alloy, hot deformation behavior, constitutive equation, processing map, DRX

## Abstract

Mg-Gd-Y-Zn-Zr Mg alloys show excellent performance in high-end manufacturing due to its strength, hardness and corrosion resistance. However, the hot deformation and dynamic recrystallization (DRX) behaviors of Mg-13.5Gd-3.2Y-2.3Zn-0.5Zr were not studied. For this article, hot compression behavior of homogenized high rare-earth (RE) content Mg-13.5Gd-3.2Y-2.3Zn-0.5Zr (wt%) alloy was investigated by using the Gleeble-3500D thermo-simulation test machine under the temperature of 350–500 °C and the strain rate of 0.001–1 s^−1^. It was found that the high flow stress corresponded to the low temperature and high strain rate, which showed DRX steady state curve during the hot compression. The hot deformation average activation was 263.17 kJ/mol, which was obtained by the analysis of the hyperbolic constitutive equation and the Zener-Hollomon parameter. From observation of the microstructure, it was found that kink deformation of long period stacking ordered (LPSO) phase was one of the important coordination mechanisms of hot deformation at low temperature. The processing map with the strain of 0.5 was established under the basis of dynamic material model (DMM); it described two high power dissipation domains: one appearing in the temperature range of 370–440 °C and the strain rate range of 0.001–0.006 s^−1^, the other appearing in the temperature range of 465–500 °C and strain rate range of 0.001–0.05 s^−1^, in which dynamic recrystallization (DRX) mainly ocurred. The highest degree of DRX was 18% from the observation of the metallographic.

## 1. Introduction

The requirements of lightweight structure and energy saving make magnesium alloy become the material selection direction of many important structural parts at present; however, the restriction of poor mechanical properties, especially the high temperature performance, affect the development of traditional magnesium alloys [1,2,3].

The alloys of Mg-Gd-Y system, which is famous for its low density, good corrosion resistance and excellent performance at both ambient temperature and elevated temperature, received more and more attention in automobile applications [4,5]. In previous research, Drits et al. developed a variety of high performance rare-earth (RE) magnesium alloys such as WE54 (Mg-5Y-1.5Nd-1.5HRE-0.5Zr), which is the most successful application of magnesium alloy [6]. After that, Rokhlin et al. found that some rare-earth elements (Gd, Dy, Tb, etc.) had greater solid solubility than Y in magnesium, and the alloy exhibited better elevated mechanical properties than WE54 alloy when the content of Gd reached 10% [7,8]. With the deepening of research, it was found that the addition of the Zn element can generate a special stacking ordered structure called LPSO phase, and the properties can be greatly improved by controlling the type and distribution of LPSO phases [9,10,11]. In the process of plastic deformation, LPSO phase coordinates deformation mainly by shearing deformation and the slipping of the non-basal system [12]. The dislocation structure of Mg_97_Zn_1_Y_2_ alloy was studied by Matsuda et al. [13], and it was found that the <a> dislocation did not locate on the basal planes while a large number of <c+a> dislocations stacked. It was believed that the LPSO structure can rise the critical shear stress (CRSS) of the slip system on the basal planes and improve the sliding of non-basal planes. Although the Mg-Gd-Y alloys exhibit highly specific performance both at room and high temperature, the applications of these alloys are still limited by the negative performance such as low machinability and elongation. Thermoplastic deformation such as extrusion is usually used to improve the properties of magnesium alloys [14,15,16].

Processing map can effectively describe the hot plastic deformation workability and determine the most reasonable thermal processing parameters, which are important to the hard deformation materials [17]. However, the Mg-Gd-Y-Zn-Zr alloy investigated in this research is a kind of alloy which is hard to deform. In previous research, Zhou et al. found that the LPSO phases can provide the back stress, which can improve thermal activation energy of materials to a high level of 359 kJ/mol [11]. 

In previous work, the microstructures with LPSO phase analysis were the key points, and mechanical properties of Mg-Gd-Y-Zn-Zr magnesium alloys have also been reported seriously [18,19,20]. However, few references of the hot deformation parameters which are important in the hot plastic deformation are available. In order to obtain the optimum hot deformation parameters of high RE content Mg-Gd-Y-Zn-Zr alloy, stress-strain curves, constitutive equation and a processing map of homogenized Mg-13.5Gd-3.2Y-2.3Zn-0.5Zr alloy were studied in this work.

## 2. Theory of Processing Map

DMM (Dynamic Materials Model) processing map is often used to describe the relationship between deformation parameters, microstructure and hot workability during hot deformation [21,22,23]. Therefore, it is one of the most important means to study the deformation microstructure and optimize the forming process. According to Von Mises [24] criterion, more than five independent slip systems are needed for polycrystalline materials to ensure that no cracks occur during the uniform plastic deformation, whereas magnesium alloys have the hexagonal close-packed hcp structure that only {0001}<11$\bar{2}$0> slip systems can move on the basal plane at room temperature, two of which are independent slip systems. When the temperature rises, the non-basal slip systems like {10$\bar{1}$0}<11$\bar{2}$0> and {10$\bar{1}$1}<11$\bar{2}$0> can be activated, and the independent slip system can be increased to eight. Therefore, high temperature deformation is a common deformation method for magnesium alloys especially for rare-earth magnesium alloys which are more difficult to deform than traditional magnesium alloys [25,26,27]. Through the establishment and analysis of the processing map, the microstructure of Mg-Gd-Y-Zn-Zr alloy can be adjusted to improve the properties of the alloy, obtain reasonable parameters, shorten the development period and reduce the cost.

The flow stress *σ* and the strain rate stress $\dot{\varepsilon }$ are related to the dynamic constitutive equation under a certain temperature [23]. The following equation is used:

(1) $$\sigma =K{\dot{\varepsilon }}^{m}$$

where “*K*” is the material constant and *m* is the strain rate sensitivity index. According to the theory of DMM, the thermal deformation material is regarded as a nonlinear energy dissipation unit. During hot deformation, the total dissipated power, *P*, is converted into two parts, one is the power *G* consumed by plastic deformation, most of which is transformed into viscoplastic energy, and a small part is stored in the form of crystal distortion energy; the other is the power *J* consumed by microstructural evolution, such as dynamic recovery (DRV), and DRX etc. Therefore, the total dissipated power is written as follows:

(2) $$P=\sigma \dot{\varepsilon }=G+J=\int_{0}^{\dot{\varepsilon }}\sigma d\dot{\varepsilon }+\int_{0}^{\sigma }\dot{\varepsilon }d\sigma$$

The proportion of dissipative power between *G* and *J* is determined by the index *m* which is sensitive to the strain rate:

(3) $$\partial J/\partial G=\partial \ln\sigma /\partial \ln\dot{\varepsilon }=m$$

Bring Equation (3) into Equation (2) and get the dissipation power *J* as follows:

(4) $$J=\frac{m}{m+1}\sigma \dot{\varepsilon }$$

Assuming that the material is in ideal linear dissipation, *J* reaches the maximum value when *m* = 1.

(5) $$J=J_{max}=\frac{\sigma \dot{\varepsilon }}{2}$$

Define $J/J_{max}$ as the dimensionless parameter, the power dissipation coefficient, *η*, can be written as follows:

(6) $$\eta =\frac{J}{J_{max}}=\frac{2m}{m+1}$$

η is an important parameter to indicate power dissipation during hot deformation. According to the dissipation coefficient, temperature and strain rate, the power dissipation map can be constructed. Generally speaking, the higher η corresponds to the better processing condition; the region with maximum dissipation coefficient η in the power dissipation map is the ideal deformation parameter range. Nevertheless, an unstable state such as micro-cracks and shear bands are also related with high η values. Prasad [28] considered the stability condition and proposed the criterion of rheological instability as follows:

(7) $$\xi \left(\dot{\varepsilon }\right)=\frac{\partial \ln\left[m/\left(m+1\right)\right]}{\partial \ln\dot{\varepsilon }}+m<0$$

where $\xi \left(\dot{\varepsilon }\right)$ represents the dimensionless parameter. The variation of $\xi \left(\dot{\varepsilon }\right)$ with deformation temperature and strain rate constructs the instable region which is characterized by $\xi \left(\dot{\varepsilon }\right)<0$. Finally, the processing map can be prepared by the overprinting of the power dissipation map and instable map, which contains two zones, the stable region and unstable region.

## 3. Materials and Methods

**Figure 1:**
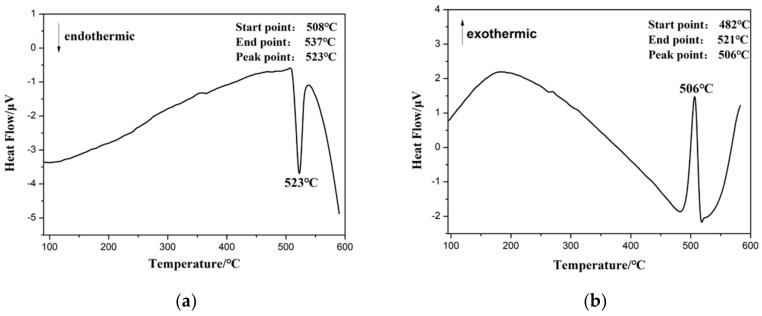
Endothermic (DSC) curve (**a**) and exothermic (DSC) curve (**b**) of the as-cast alloy.

The magnesium alloy of Mg-13.5Gd-3.2Y-2.3Zn-0.5Zr (wt%) with 420 mm in diameter was casted by semi-continuous casting. Then, cylindrical samples with the size of Φ8 mm × 12 mm were machined from the Mg alloy ingot. The eutectic phase transition temperature of the as-cast magnesium alloy was calculated to be 523 °C (as shown in Figure 1) by differential scanning calorimetry (DSC, Q20, TA, New Castle, PA, USA). The alloys were homogenized at 515 °C for 20 h—which has the least number of the bulk-shaped LPSO phases that affect the deformation. Finally, the ~70 °C water quenching was used.

**Figure 2:**
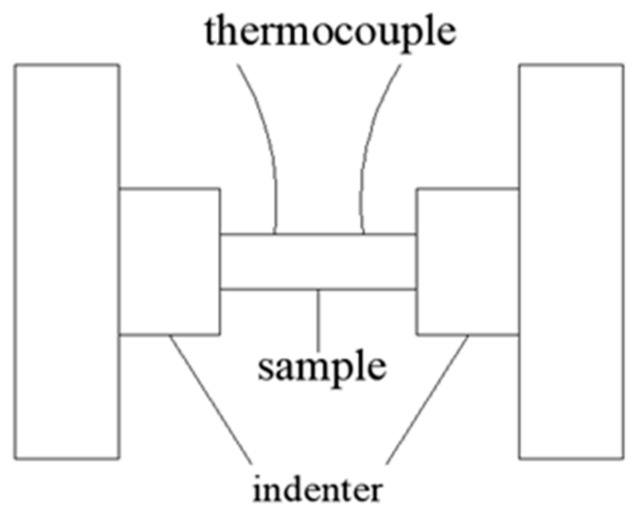
Schematic diagram of hot compression.

Uniaxial compression tests were carried out under the temperature of 350–500 °C and the strain rate of 0.001–1 s^−1^ on the Gleeble-3500D thermo-simulation test machine (3500D, Dynamic Systems Inc., New York, NY, USA) and graphite sheet were used as lubricant before compression and placed between the indenter and the sample, which was used to reduce the deformation friction (as shown in Figure 2). Then, the samples were heated with a heating rate of 10 °C/s by using the resistance heating method and held for 5 min. The true stress-strain curves were obtained from the machine by using its analysis system. After the deformation, the samples were quenched into the water immediately to keep the high temperature microstructure. Before observation, the metallographic specimens were ground, polished and chemically corroded in a solution of 4.5 g picric acid, 70 mL ethanol, 10 mL 99% acetic acid and 10 mL deionized water.

The phase composition and atomic ratio were measured by using the X-ray diffractometer (XRD, DX-2700, Fangyuan Inc., Dandong, China) with the parameter of diffraction angle from 25° to 85° and scanning speed of 5°/min. Then, the microstructures of the compressed specimens were observed by optical microscope (OM, A1m, Zeiss, Oberkochen, Germany) with the magnification range of 50–500. The scanning electron microscopy (SEM, SU5000, Hitachi, Tokyo, Japan) was used to observed the phase morphology with the voltage of 20 kV. Meanwhile, the energy dispersive X-ray spectrometer (EDS, Genesis, EDAX Inc., Mahwah, NJ, USA) and electron backscattered diffraction (EBSD, EDAX Inc., Mahwah, NJ, USA) were operated to define the phase atomic ratio and grain orientation at 20 kV, title angle of 70°, and working distance of 15 mm. Last, the EBSD data were processed by the TSL OIM software (version 7.3, EDAX Inc., Mahwah, NJ, USA).

## 4. Results and Discussion

### 4.1. Microstructure of As-Cast and Homogenized Samples

**Figure 3:**
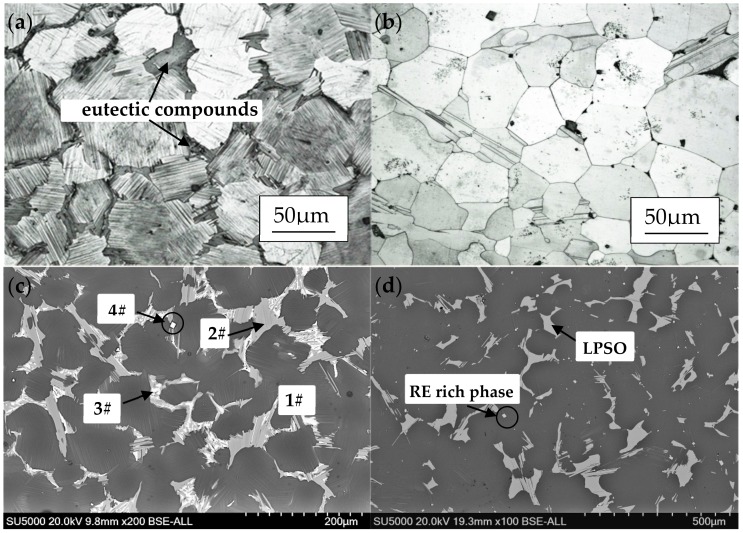

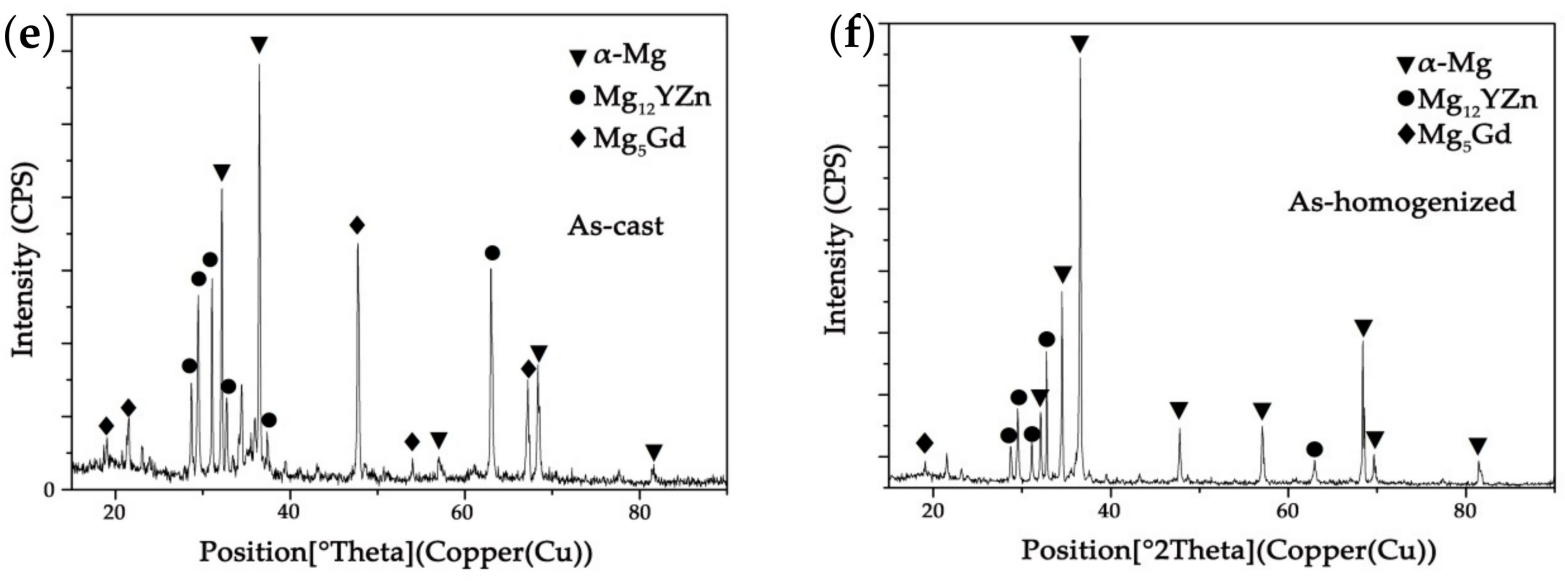
Microstructure observation of (**a**,**c**) as-cast alloy, (**b**,**d**) homogenized alloy and XRD patterns of Mg alloy before (**e**) and after (**f**) homogenization.

The initial microstructure, as-homogenized microstructure and the XRD patterns of Mg-13.5Gd-3.2Y-2.3Zn-0.5Zr alloy are depicted in Figure 3. As seen, the as-cast alloy is mainly composed of α-Mg matrix and the eutectic compounds which precipitate at the grain boundaries; the average grain size is 65 μm. The volume fraction of the eutectic compounds decreased after the homogenization of 515 °C × 20 h and the bulk-shaped phases precipitated at the confluence of boundaries as shown in Figure 3b. The XRD studies of as-cast and homogenized alloys confirm that α-Mg phase, Mg_12_RE_1_Zn_1_ and Mg_5_(Gd,Y) are the constituent of the target Mg alloy. Figure 3c shows the SEM micrograph of the as-cast alloy and the element atomic percentage obtained from EDS is given in Table 1; it is obviously seen that the discontinuous distribution block-shaped phases have different contrasts which are marked 2# and 3#, respectively, and discrete distribution bright white square 4# also attracts attention. Then, the XRD and EDS studies (as shown in Table 1) of Mg alloy confirm that the 1# is α-Mg matrix. The chemical equation of 2# in the eutectic compound is Mg-4.21Gd-2.62Y-5.03Zn whose ratio of Gd+Y to Zn is close to 1:1, and the stoichiometry is close to Mg_12_RE_1_Zn_1_. In addition, the chemical equation of the bright phase 3# is Mg-11.35Gd-4.32Y-2.10Zn and the stoichiometry is close to Mg_5_(Gd,Y). In the previous work, the approximate chemical composition of 14H LPSO structure in Mg_96.5_Zn_l_Gd_2.5_ alloy is Mg-11Zn-8Gd (at.%), and its Zn/Gd atom ratio is about 1.17:1 [13], so it is speculated that the LPSO phases have been formed in the microstructure of as-cast alloy, and the 14H type LPSO structure of Zn/RE in Mg-Zn-Gd alloy is maintained [29,30]. At last, the bright white structure 4# mainly contains a large amount of Y, Gd and a small amount of Mg, Zr, and quite a small amount of Zn, which is confirmed to be the rich rare-earth phase. Obviously, the bright phases in the eutectic compounds dissolved and transformed into the dark phases, which are shown in the SEM diagrams, and the XRD diagram confirms the decrease of Mg_5_(Gd,Y) phases and the increase of Mg_12_RE_1_Zn_1_.

### 4.2. Compression Behavior and Microstructure Evolution

#### 4.2.1. Flow Stress Behavior

**Figure 4:**
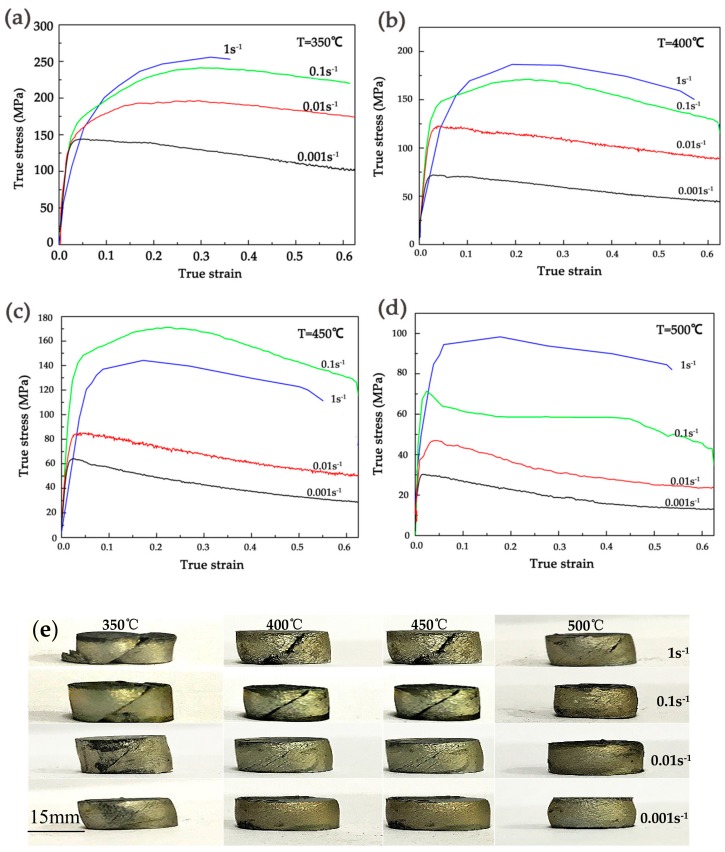
True stress-strain curves of the homogenized alloy compressed at the temperature of (**a**) 350 °C, (**b**) 400 °C, (**c**) 450 °C, and (**d**) 500 °C; (**e**) photographs of the samples after extrusion.

**Figure 5:**
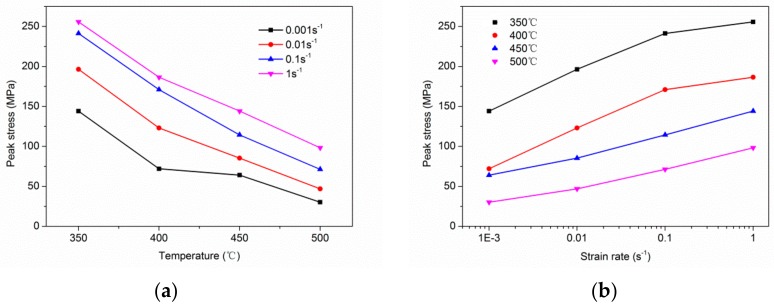
Relationship among flow stress, (**a**) deformation temperature and (**b**) strain rate.

The variations of stress with strain of the homogenized alloys at the temperatures of 350 °C, 400 °C, 450 °C and 500 °C and strain rates 0.001/s, 0.01/s, 0.1/s and 1/s are presented in Figure 4a–d. Obviously, the flow stress increases with the decrease of temperature and the increase of strain rate, this trend is noticeably shown in Figure 5. There are three stages in the true stress-strain curves: Firstly, due to the deformation hardening, the flow stress increases sharply to a relatively high state but not the maximum. Secondly, the stress increases to the maximum (peak stress) slightly, which was the result of competition between deformation hardening and dynamic recrystallization (DRX) softening. Thirdly, with the increase of strain rate, the flow stress decreases slightly. Further research tells that: (1) The flow steady state is easier to achieve and the deformation hardening is weaker at low strain rate ($\dot{\varepsilon }$ = 0.001/s) than the high strain rate ($\dot{\varepsilon }$ = 1/s), and it causes the peak stress at the lower strain rate to be lower than the higher strain rate at a given temperature. (2) The stress-strain curves are not complete when the strain rate is 1/s, early failures happened at the strain rate higher than 1/s, especially at 350 °C, which means the deformation was dominated by the work hardening, and it is difficult to deform at or higher than 1/s. (3) According to the compression tests shown in Figure 5, because of the dislocation storage and stress concentration, the higher strain rate and the lower temperature are both not the optimum deformation parameter. (4) It can obviously be seen that the curves show dynamic recrystallization (DRX) characteristics when the compression process happened at the strain rate of 0.01/s and 0.001/s at a given temperature, and the stress-strain curves have typical peak values and steady state phenomena, which is due to the fact that the stacking fault energy of the magnesium alloy can be lower and the width of the extension dislocation is wider. (5) Thermal deformation of magnesium alloys is a process of competition between the deformation hardening and the thermally activated softening effect of DRX [31]. As shown in Figure 4e, it confirms that the low deformation temperature and high strain rate would make the cracks extend, which reveals the poor machinability of the alloy under that condition.

#### 4.2.2. Effect of Temperature

**Figure 6:**
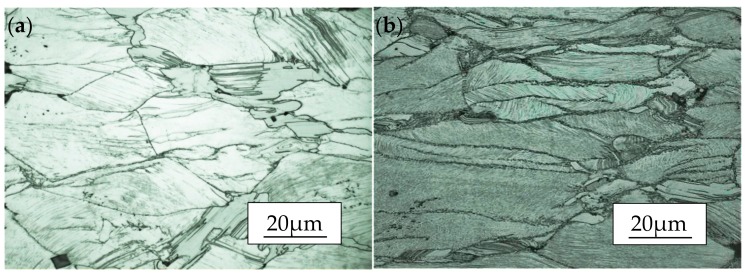

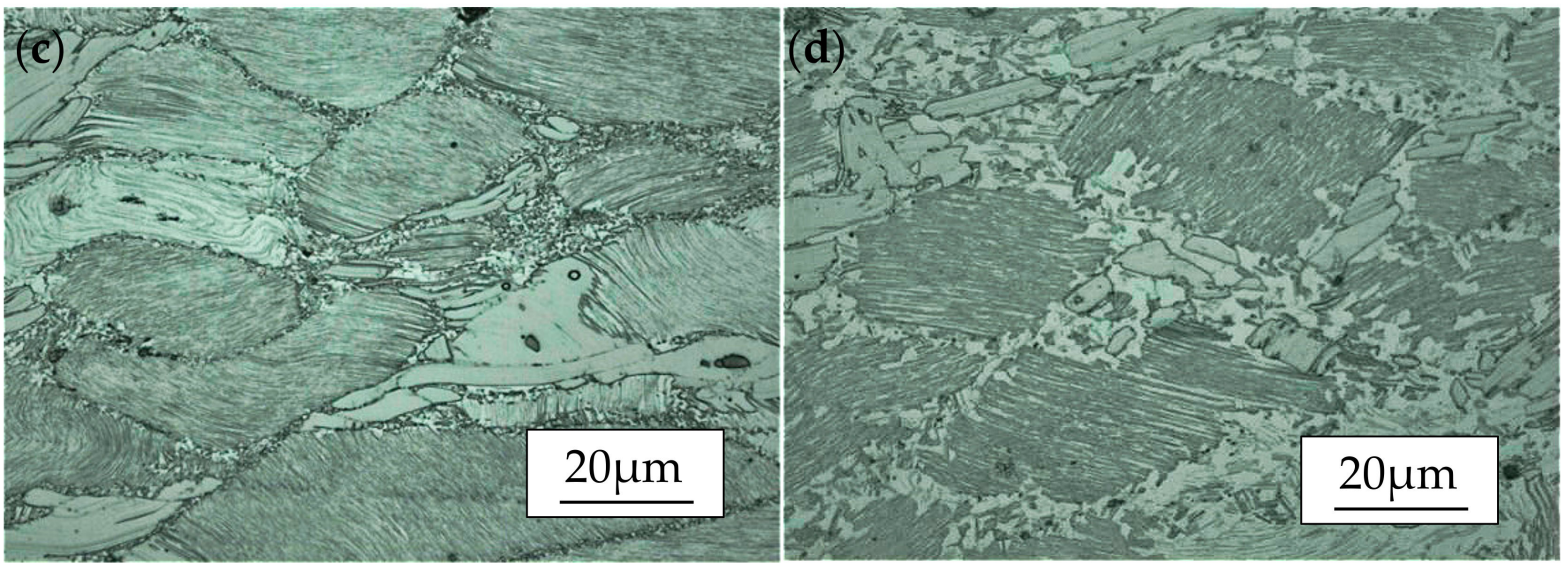
Microstructure image of the Mg-13.5Gd-3.2Y-2.3Zn-0.5Zr alloy compressed at the rate of 0.01/s and processing temperature of (**a**) 350 °C, (**b**) 400 °C, (**c**) 450 °C, and (**d**) 500 °C.

The hot deformation microstructures are illustrated in Figure 6 for samples at the strain rate of 0.01/s deformed at 350, 400, 450 and 500 °C. It can be observed that the DRX occurs at the temperature of 400–500 °C and the DRX cannot be obviously seen at 350 °C, and the grains are elongated along the direction perpendicular to the compression. As can be seen, the area fraction of DRX is relatively low at the given temperatures, the fine lamellar-shaped LPSO phases which are unevenly distributed show different orientations, corresponding to some of them undergo the kink deformation and form the kink bands. During the deformation at low temperature, the condition of recrystallization nucleation cannot be reached completely, resulting in a small number of DRX grains distributing at the grain boundaries, which shows a dDRX process. As the temperature increases to 400 °C (as shown in Figure 6b), it is obviously seen that the volume fraction of DRXed grains is a little bit higher, and the “necklace” structure that the fine grains gather around the coarse grains becomes prominent. As the temperature rises to 450 °C (shown in Figure 6c), recrystallization grains grow slightly and the area fraction of precipitated particles decreases a little, indicating the not suitable deformation temperature. At 500 °C (as shown in Figure 6d), enough thermal energy is the key point to promote growth of the recrystallization grains which mainly occur at the serrated grain boundaries. Meanwhile, the number of particulate precipitates significantly decreased. Therefore, the temperature of 450 °C is chosen for further microstructure observation at different rates.

According to the comparison of Figure 6a–d, it is worth noticing that the contrast of fine lamellar-shaped LPSO phases deepen with the increase of temperature, indicating that precipitation of lamellar-shaped LPSO phases can be led by hot compression. Meanwhile, it can be known that two conditions are required for the formation of lamellar-shaped LPSO phases, one is structure, where stacking faults with crystal defects are needed; and the other is composition, where alloy elements meet a certain proportion. Also, the formation of lamellar-shaped LPSO phases in grains is a diffusion-dominated process, in which the rate of atomic diffusion is accelerated with the increase of temperature which is conductive to the precipitation of the lamellar-shaped LPSO phase. In addition, the dislocations and other defects can reduce the diffusion activation energy to a certain extent and accelerate the diffusion of solute atoms. Generally, both high temperature and deformation contribute to the formation of the lamellar-shaped LPSO phases. Otherwise, because of the stability of the hexagonal close-packed (hcp) structure, kink deformation is an important method to coordinate deformation when the basal slip is difficult to activate [32]. The lamellar-shaped LPSO phases hinder the growth of DRX grains and the area fraction of recrystallized grains shows a low state where the grains are fine.

#### 4.2.3. Effect of Strain Rate

**Figure 7:**
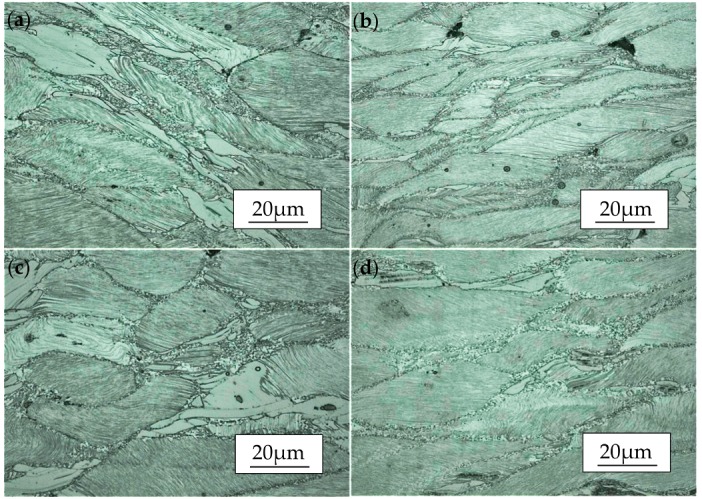
Microstructure of the Mg-13.5Gd-3.2Y-2.3Zn-0.5Zr alloy compressed at the temperature of 450 °C and strain rate of (**a**) 1/s, (**b**) 0.1/s, (**c**) 0.01/s, and (**d**) 0.001/s.

Microstructure of the compressed specimens at the temperature of 450 °C is shown in Figure 7. Compared with Figure 6, the contrast of lamellar-shaped LPSO phases shown in Figure 7 with different strain rates do not change significantly, indicating that the effect of temperature on the precipitation of lamellar-shaped phases is stronger than that of strain rate. Figure 7a shows the compressed microstructure of samples at the rate of 1/s. As seen, the volume fraction of DRXed grains is low, which means the dynamic recrystallization is not complete yet. During the compression, the coarse grains were extruded and elongated, accompanied with the kink deformation of the LPSO phases. Meanwhile, it can be noticed that shear bands are formed by local severe plastic deformation, and the DRX occurs mainly around them. With the strain rate decrease to 0.1/s which is shown in Figure 7b, it can be seen that the fine lamellar-shaped LPSO phases maintain different orientations with increased kink bands and there are more fine DRX grains at the grain boundaries. The slowing down of deformation velocity is beneficial to the progress of DRX [33], and the effect of DRX on the release of stress concentration at the grain boundary is enhanced. Strain rates of 0.01/s and 0.001/s are shown in Figure 7c,d respectively, and it can be observed that the area fraction and grain size of DRX hardly changed due to the blocking of the bulk-shaped LPSO phase to grain boundary migration. In general, from Figure 7a–d, the storage energy of material increases with the increase of dislocation accumulation, corresponding to improving the recrystallization nucleation rate with an increase of the strain rate at a given temperature. Meanwhile, because of the short deformation response time, the DRX grains cannot grow up timely and the recrystallized grain size is relatively small [34,35,36]. Also, it is difficult to slip for dislocation because of the existence of the LPSO phase, which accompanies the inhibition of nucleation and growth of DRX, leading to the low level of recrystallization volume fraction [25]. Meanwhile, the increase of dislocation density causes the formation of more kink bands, which provide a favorable position for dynamic recrystallization nucleation. Therefore, it is foreseeable to see the increase of nucleation in DRX. In summary, fine recrystallized grains with a “necklace” structure are produced at the grain boundary and at the kink bands with the condition of low strain rate like 0.001/s; however, if the strain rate is too high, it will increase the degree of irregular deformation.

### 4.3. Kinetic Analysis

For investigating the hot workability behavior of Mg-13.5Gd-3.2Y-2.3Zn-0.5Zr alloy, it is significant to study the constitution equation [37], which is a mathematical model describing the relationship between the deformation parameters, such as temperature, stress and strain, and strain rate which are of great significance for understanding the deformation mechanism for the material [38].

The deformation of metal materials at high temperatures is a process determined by thermal activation, satisfying the Arrhenius Equation [39]:

(8) $$\dot{\varepsilon }==A{\left[\sinh\left(\alpha \sigma \right)\right]}^{n}\exp\left(-\frac{Q}{RT}\right)$$

The relationship between strain rate $\dot{\varepsilon }$ and the steady flow stress *σ* at low stress level (*ασ* < 0.8), high stress level (*ασ* > 1.2) and the whole stress range can be expressed as follows [40]:

(9) $$\dot{\varepsilon }=A\sigma^{n}\exp\left(-\frac{Q}{RT}\right)$$

(10) $$\dot{\varepsilon }=A\exp\left(\beta \sigma \right)\exp\left(-\frac{Q}{RT}\right)$$

where *Q* is the DRX activation energy (kJ/mol), *T* is the absolute temperature (K), *R* is gas constant (8.314 J/mol·K) and $\dot{\varepsilon }$ is the strain rate. *A* (*A*_1_, *A*_2_), *α*, *β* and *n* are the general constants that are related to the materials, with *α* = *β*/*n*_1_ [18] and *σ* is the peak stress or steady stress (MPa).

The logarithm of Equation (9) and Equation (10) can be obtained as follows:

(11) $$\ln\dot{\varepsilon }=\ln A_{1}-Q/RT+\beta \sigma$$

(12) $$\ln\dot{\varepsilon }=\ln A_{2}-Q/RT+n_{1}\ln\sigma$$

**Figure 8:**
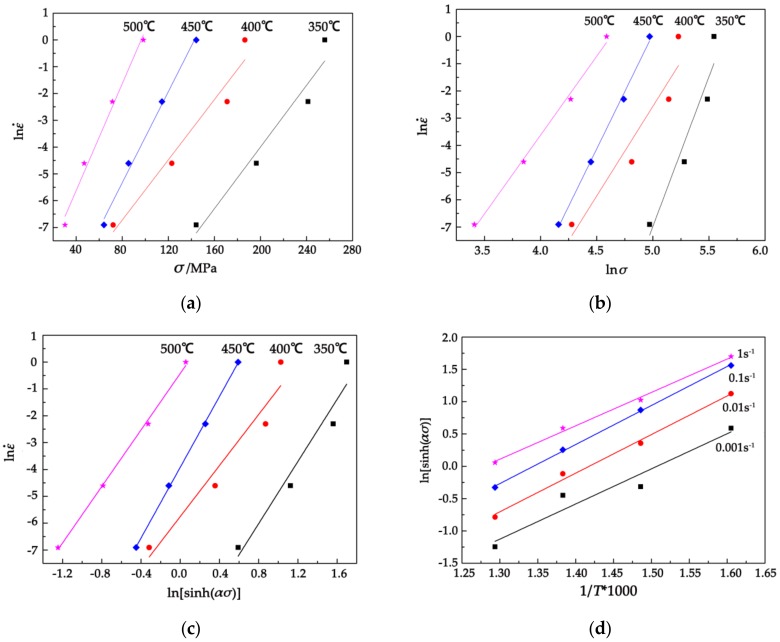
Plots used for the calculation of hot deformation constants (**a**) $\ln\dot{\varepsilon }-\ln\sigma$; (**b**) $\ln\dot{\varepsilon }-\sigma$; (**c**) $\ln\dot{\varepsilon }-\ln\left[\sin h\left(\alpha \sigma \right)\right]$; and (**d**) ln[sinh(ασ)]−(1000/T).

Figure 8a,b shows the relationship between the peak stress and strain rate in which Equations (11) and (12) are present. It can be noticed that the relationship between the peak stress $\sigma_{P}$ and the strain rate $\dot{\varepsilon }$ is linear and the correlation coefficients of the fitted lines are more than 0.97 and 0.95. The mean value of slopes with low stress level and high stress level are taken as *n* and *β*, respectively. The calculating data can be obtained by the slope of the curves in Figure 8a,b, *n*_1_ = 7.954 and *β* = 0.075, respectively, so $\alpha =\beta /n_{1}=9.381\times 10^{-3}{\;\mathrm{mm}}^{2}/N$.

Figure 8c,d show the diagram of $\ln\dot{\varepsilon }-\ln\left[\sin h\left(\alpha \sigma \right)\right]$ and ln[sinh(ασ)]−(1000/T). It can be seen that the linear correlation coefficients are greater than 0.96 by performing linear regression analysis and the slope of the straights are not the same. The activation energy *Q* is influenced by deformation parameters such as temperature and strain rate. The dislocation density increases with the strain increasing and the work hardening makes the deformation difficult, so the greater activation energy is needed. While at a high temperature, the dislocation is easy to slip and climb and the dynamic recrystallization is produced which makes the activation energy reduce accordingly. When the strain is high, the dislocation density increases rapidly and the dynamic recrystallization is low, so the slip of the dislocation requires a larger deformation activation energy. The average linear slope of $\ln\dot{\varepsilon }-\ln\left[\sin h\left(\alpha \sigma \right)\right]$ (shown in Figure 8c) is 5.60 when the temperature is constant. And when the strain is constant, the average linear slope of ln[sinh(ασ)]−(1000/T) is 0.57. The average deformation activation energy at different temperatures and strain rates is *Q* = 263.17 kJ/mol.

The larger the activation energy, the more difficult the deformation is; the dynamic precipitation [41], pinning effect and second equivalence can hinder the dislocation motion, meanwhile, the deformation activation energy increases. It can be calculated that the activation energy of homogenized Mg-Gd-Y-Zn-Zr alloy is 263 kJ/mol which is much larger than that of pure magnesium 135 kJ/mol. Compared with conventional commercial magnesium alloys, the activation energy of homogenized alloy is much higher which indicates that the LPSO phase and lamellar structure can effectively hinder the movement of dislocations and leads to its higher activation energy.

Thermal deformation conditions are usually described by temperature compensated Zener-Hollomon (*Z*) [42].

(13) $$Z=\dot{\varepsilon }exp\left(Q/RT\right)=A{\left[\sin h\left(\alpha \sigma \right)\right]}^{n}$$

(14) $$\sin\left(\alpha \sigma \right)={\left(Z/A\right)}^{n}$$

(15) $$\sin h^{-1}\left(\alpha \sigma \right)=\ln{\left(\alpha \sigma +\alpha \sigma^{2}+1\right)}^{1/2}$$

According to the definition of hyperbolic sine function, it can be seen that the relation between *σ* and *Z* is as follows:

(16) $$\ln Z=\ln\dot{\varepsilon }+\left(Q/RT\right)=n\ln\left[\sin h\left(\alpha \sigma \right)\right]+\ln A$$

From Equation (16), the deformation activation energy *Q* can be obtained as follows:

(17) $$Q=R{\left\{\frac{\partial \ln\dot{\varepsilon }}{\partial \ln\left[\sin h\left(\alpha \sigma \right)\right]}\right\}}_{T}\cdot {\left\{\frac{\partial \left[\mathrm{lnsin}h\left(\alpha \sigma \right)\right]}{\partial \left(1/T\right)}\right\}}_{\dot{\varepsilon }}$$

**Figure 9:**
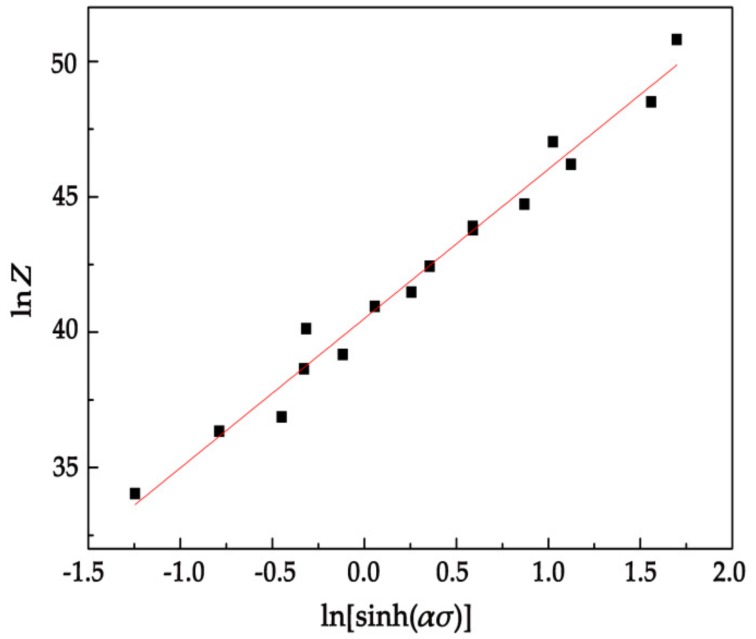
Relationship between the Zenner-Hollomon parameter: lnZ−ln[sinh(ασ)].

Figure 9 shows the lnZ−ln[sinh(ασ)] plots using the least-square method, *n* and lnA are calculated to be 5.514 and 40.504, respectively. At the same time, the value of *A* can be calculated as 3.897 × 10^17^ s^−1^. The constitutive equation of the homogenized Mg-13.5Gd-3.2Y-2.3Zn-0.5Zr alloy can be obtained as follows:

(18) $$\dot{\varepsilon }=3.897\times 10^{17}{\left[\sin h\left(9.381\times 10^{-3}\sigma \right)\right]}^{5.514}exp\left(-\frac{263165}{8.314T}\right)$$

Furthermore, substitute the value of *α*, *n* and *Q* to Equation (16), the constitutive equation of homogenized Mg-13.5Gd-3.2Y-2.3Zn-0.5Zr alloy at elevated temperature can be expressed by:

(19) $$\sigma =106.598\left\{{\left(\frac{Z}{3.897\times 10^{17}}\right)}^{\frac{1}{5.5143}}+{\left[{\left(\frac{Z}{3.897\times 10^{17}}\right)}^{\frac{2}{5.5143}}+1\right]}^{\frac{1}{2}}\right\}$$

### 4.4. Characterization of Processing Map and Microstructure of Stability and Instability Regions

**Figure 10:**
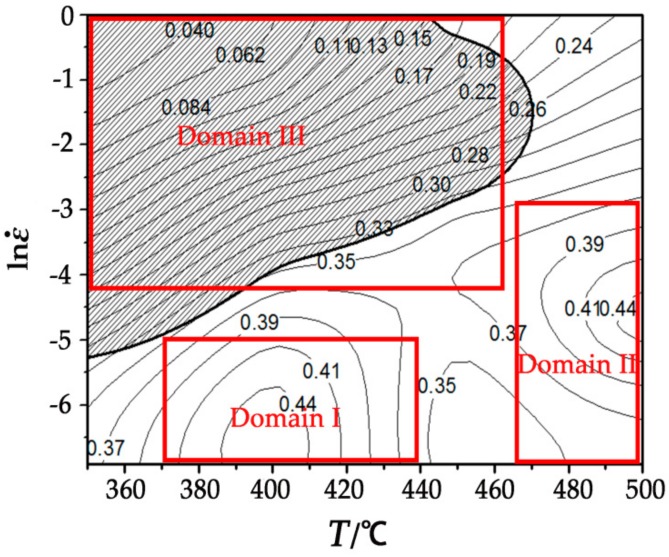
Processing map with the strain of 0.5.

Figure 10 shows the processing map with the strain rate of 0.5. The contour value represents the efficiency of power dissipation and the shade area reflects regions of flow instability. It can be noticed from the processing map that deformation rate is the important factor affecting the power dissipation factor. The flow instability region is distributed mainly in the high strain rate and low temperature region. As known, the safe conditions are mainly concentrated on the high power dissipation, and there are two high power dissipation regions in the stability region which are domainI (370–440 °C/0.001–0.006 s^−1^) and domainII (465–500 °C/0.001–0.05 s^−1^) with the value of about 44%.

**Figure 11:**
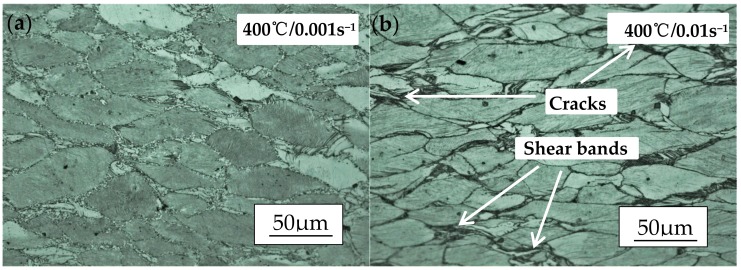

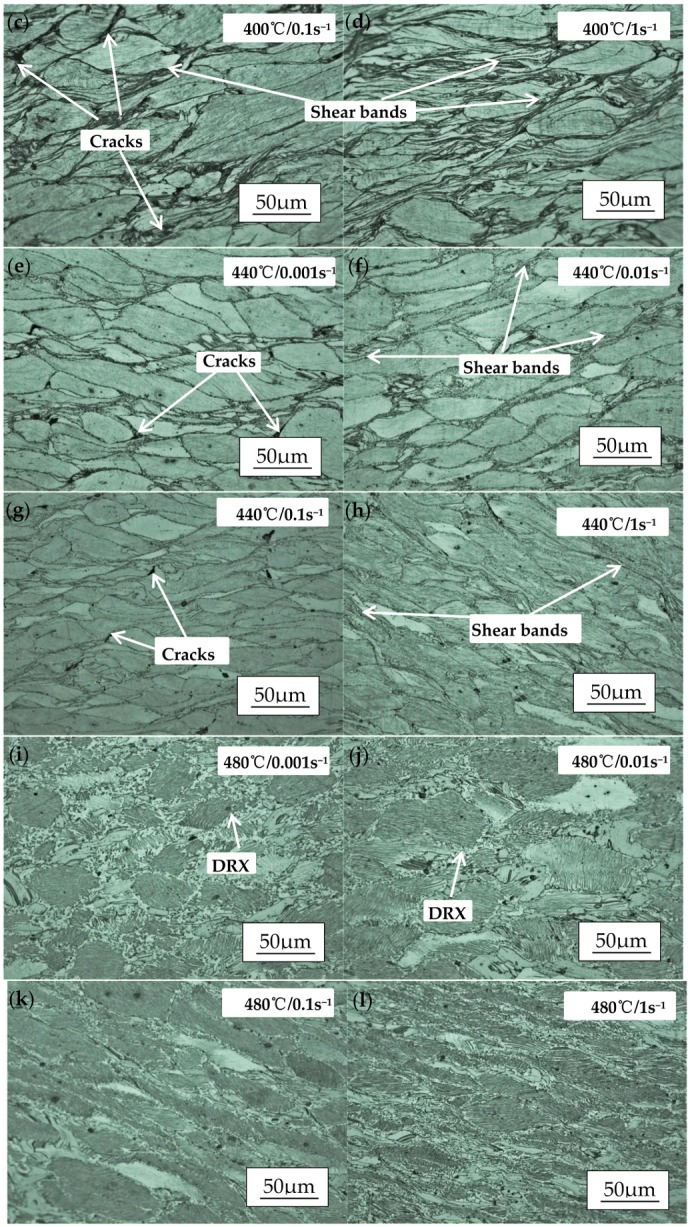
Micrographs of Mg-13.5Gd-3.2Y-2.3Zn-0.5Zr alloy compressed at 400 °C, 440 °C and 480 °C at the strain rate of 0.001/s, 0.01/s, 0.1/s and 1/s.

**Figure 12:**
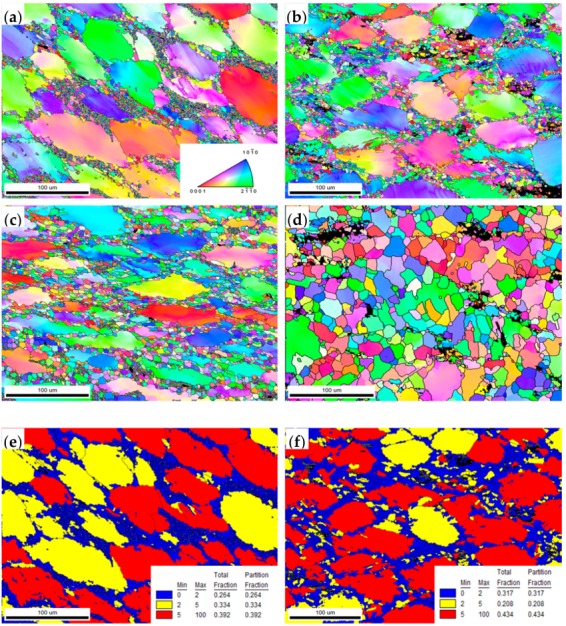

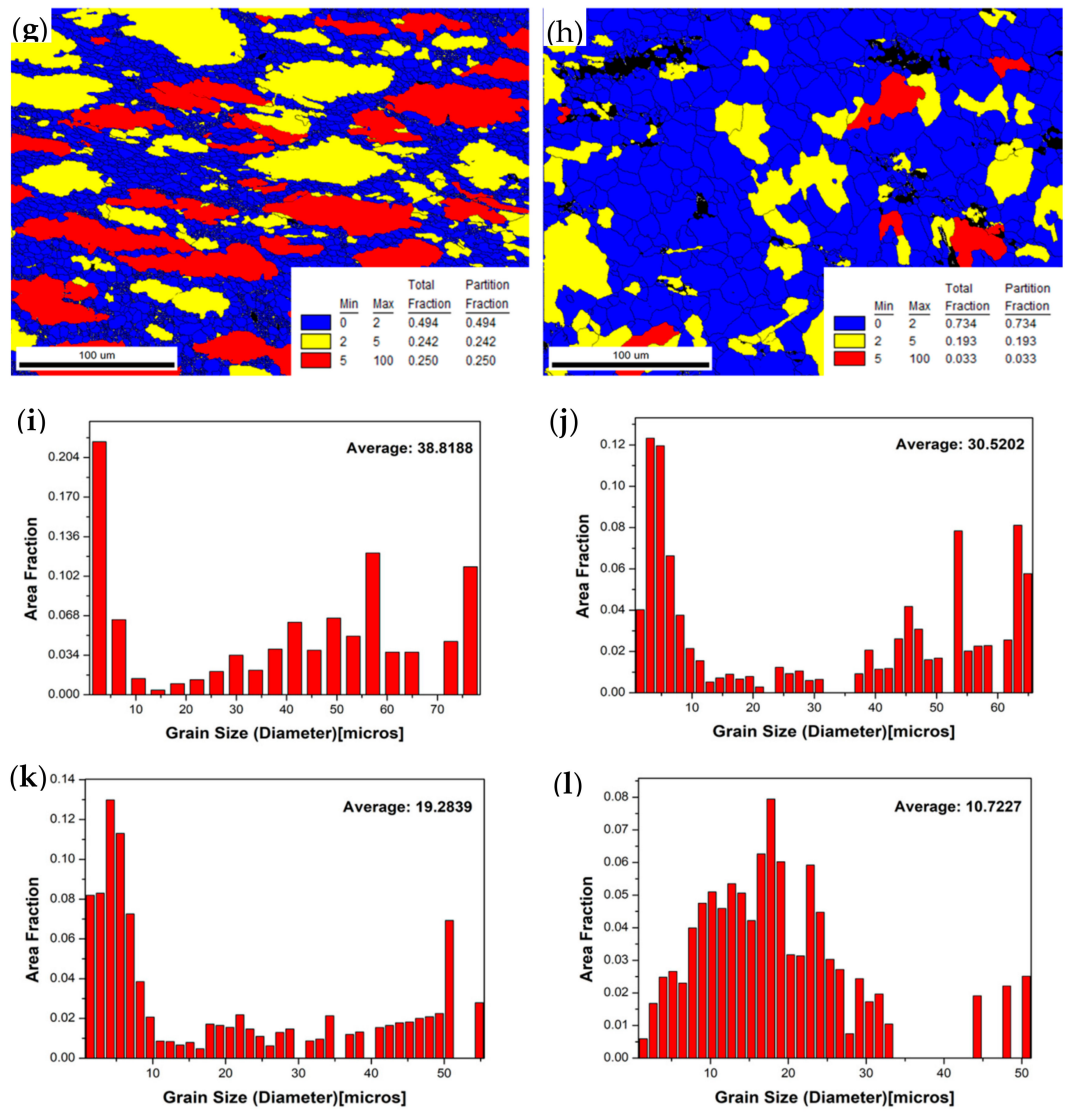
The IPF maps (**a**–**d**), DRX ratio maps (**e**–**h**) and average grain size maps (**i**–**l**) of compressed samples under different conditions: (**a**,**e**,**i**) 400 °C/0.01 s^−1^, (**b**,**f**,**j**) 450 °C/0.01 s^−1^, (**c**,**g**,**k**) 500 °C/0.01 s^−1^, and (**d**,**h**,**l**) 500 °C/0.001 s^−1^.

Based on the discussion above, both stability and instability domains are shown in Figure 11. It is easy to see that the DRX happened among them, while the degrees vary a lot. The LPSO phases are elongated to align perpendicular after compression and the DRX occurs that many fine recrystallization grains around the coarse grains. The DRX ratio increases with the rise of temperature and the reduce of strain rate, but the relatively complete dynamic recrystallization happens at 480 °C with the strain rate of 0.001/s (as shown in Figure 12i). According to the high power dissipation region obtained from the processing map, it can be measured that the strain rate takes the significant role at stability domain I which is shown in Figure 10. The studies of Figure 11a–d confirm that the adiabatic shear bands and micro-cracks seriously affect the processing performance of materials as the strain rate is higher than 0.001/s. Meanwhile, the DRX ratio stays at a low level because of the low temperature. As the compression temperature increases to 440 °C, defects such as adiabatic shear bands and micro-cracks reduce to a low level, but the DRX does still not reach a relatively high level. Under most conditions, the poor workability of the alloy can be associated with the bimodal microstructure. Figure 11i–l shows the microstructure of 480 °C at different strain rates. It is obvious that adiabatic shear bands do not occur which is due to the sliding system activation, the further increase of the DRX ratio and the torsional deformation of LPSO phase is no longer necessary. Thus, 480 °C/0.001 s^−1^ is the optimum stable parameter for homogenized Mg-Gd-Y-Zn-Zr alloy.

Figure 12 shows the IPF maps, dynamic recrystallization ratio and average size maps of the samples compressed at the temperatures of 400 °C, 450 °C and 500 °C and the strain rate of 0.01/s and 0.001/s. It can be seen that the DRX ratio improves with the increase of temperature, the decrease of strain rate and the DRX complete at 500 °C/0.001 s^−1^. The black regions represent the block-shaped LPSO phases without the sufficient confidence index (CI) and the blue regions shown in Figure 12e–h represent the DRX grains. Obviously, the DRXed grains distribute at the original grain boundaries (as shown in Figure 12a–d) and the DRX ratios are 26%, 31%, 49% and 73%, respectively. As seen in Figure 12i–l, the average grain sizes of the compressed samples of different temperatures and strain rates are 38 mm, 30 mm, 19 mm and 10 mm. As the main softening mechanism, the occurrence of DRX lowers distortion inside the coarse unDRXed grains; the DRX ratio is important evidence for choosing the deformation parameters, so it makes the 500 °C/0.001 s^−1^ a considerable one.

## 5. Conclusions

In this research, the hot deformation behavior of homogenized Mg-13.5Gd-3.2Y-2.3Zn-0.5Zr alloy via hot compression tests has been studied at the temperature from 350 °C to 500 °C and the strain rate range of 0.001–1 s^−1^. Based on true stress-strain curves, the constitutive equation, the processing map, and deformation of RUE to verify the parameters, the conclusions are as follows:

(1) The homogenized alloy exhibits the typical dynamic recrystallization behavior characteristics. The flow stress increases with the decrease of temperature and the increase of strain rate. Meanwhile, the true stress-strain curves tell that the high strain rate and low temperature make a high work hardening rate.
(2) The constitutive equation of the homogenized alloy is $\dot{\varepsilon }=3.897\times 10^{17}{\left[\sin h\left(9.381\times 10^{-3}\sigma \right)\right]}^{5.514}\exp\left(-263165/8.314T\right)$ and the deformation activation energy Q (~263.15 kJ/mol) of homogenized alloy is much higher than that of pure Mg (~135 kJ/mol), which is due to the existence of LPSO phases.
(3) LPSO kink deformation becomes an important mean to coordinate deformation when it is difficult to start the sliding system of magnesium alloy during deformation at low temperature. From the further observation of the microstructure evolution, the DRX softening enhances with the increase of temperature which makes the slip system easy to slide. Meanwhile, the flow instability and the stress concentration become serious and the micro-cracks are produced at the intersection of kinks where the lump LPSO phases gather.
(4) According to the processing map, dynamic recrystallization behavior with the strain of 0.5 occurs at two high power dissipation zones: 370–440 °C/0.001–0.006 s^−1^ and 465–500 °C/0.001–0.05 s^−1^, respectively, which correspond to the peak power dissipation of 44%. From the observation of compressed specimens, the optimum condition for hot plastic deformation is 465–500 °C/0.001–0.05 s^−1^.

## Figures and Tables

**Table 1:** The element atomic percentage of marked points in Figure 3c.

Points	Mg	Gd	Y	Zn	Zr
1#	98.20	0.55	0.23	0.80	0.22
2#	88.03	4.21	2.62	5.03	0.11
3#	81.89	11.35	4.32	2.10	0.34
4#	29.10	24.83	42.51	0.71	2.85
